# The hemoglobin glycation index predicts the risk of adverse cardiovascular events in coronary heart disease patients with type 2 diabetes mellitus

**DOI:** 10.3389/fcvm.2022.992252

**Published:** 2022-11-03

**Authors:** Shuai Xu, Zhen Qin, Ruixia Yuan, Xiaolin Cui, Li Zhang, Jing Bai, Gangqiong Liu, Zeyu Wang, Fengyi Yu, Yan Lv, Jinying Zhang, Junnan Tang

**Affiliations:** ^1^Department of Cardiology, The First Affiliated Hospital of Zhengzhou University, Zhengzhou, China; ^2^Henan Province Key Laboratory of Cardiac Injury and Repair, Zhengzhou, China; ^3^Henan Province Clinical Research Center for Cardiovascular Diseases, Zhengzhou, China; ^4^Clinical Big Data Center, The First Affiliated Hospital of Zhengzhou University, Zhengzhou, China; ^5^School of Medicine, The Chinese University of Hong Kong, Hong Kong, Hong Kong SAR, China; ^6^Department of Bone and Joint, The First Affiliated Hospital of Dalian Medical University, Dalian, China

**Keywords:** coronary heart disease, type 2 diabetes, hemoglobin glycation index, glycated hemoglobin, major adverse cardiovascular events

## Abstract

**Background:**

Previous studies have shown that the hemoglobin glycation index (HGI) can be used as a predictor of diabetic complications. However, limited information is currently available to indicate the correlation between HGI and comorbidity of coronary heart disease (CHD) and diabetes. This study aimed to evaluate the potential of HGI to predict major adverse cardiovascular events (MACEs) in CHD patients with type 2 diabetes mellitus (T2DM) undergoing percutaneous coronary intervention (PCI).

**Materials and methods:**

A total of 918 CHD patients with T2DM were enrolled in a 3-year retrospective cohort study, from December 2017 to December 2020 at the First Affiliated Hospital of Zhengzhou University. Data including fasting blood glucose (FPG/FBG) and glycated hemoglobin A1c (HbA1c) were collected. HGI was calculated as actual measured HbA1c minus predicted HbA1c. Three groups were further divided based on the levels of HGI, including low, medium, and high levels.

**Result:**

Kaplan Meier analysis indicated that elevated HGI was strongly associated with the occurence of MACE (log-rank *P* < 0.001). Multivariate Cox regression analysis revealed that elevated HGI was an independent risk factor for incident MACE in CHD patients with T2DM [adjusted hazard ratio (HR): 1.473; 95% confidence interval (CI): 1.365-1.589, *P* < 0.001].

**Conclusions:**

Hemoglobin glycation index is an independent predictor of MACE events in CHD patients with T2DM. High HGI indicates a higher risk of MACE occurrence.

## Introduction

Despite the recent COVID-19 pandemic, cardiovascular disease (CVD) remains the leading cause of morbidity and mortality worldwide (1). Coronary heart disease (CHD), also known as ischemic heart disease, is a common CVD caused by coronary artery atherosclerosis, vasospasm-stenosis, or complete occlusion, impeding normal myocardial blood supply (1). The pathophysiological mechanisms of CHD mainly include lipid metabolism disorders, microvascular dysfunction, endothelial dysfunction, inflammation and immune response, oxidative stress, etc. Some factors such as obesity, smoking and alcohol consumption significantly contribute to the development of CHD (2, 3). In addition, other risk factors, including hypertension, hyperlipidemia, hypercholesterolemia, diabetes mellitus, also lead to CHD (4, 5). It is important to note that type 2 diabetes is a major risk factor. Hyperglycemia in patients with type 2 diabetes often promotes oxidative stress and inflammatory damage, which directly leads to atherosclerosis, coronary insufficiency, myocardial infarction, and cell damage, resulting in the development of CHD (6). Unsurprisingly, most diabetic patients also have CHD, with poor prognosis (7). With the changes in living conditions and diet structure, it is expected an increasing number of type 2 diabetes and CHD patients (8). According to the “2019 ESC Guidelines” and “2020 ASCVD Chinese Consensus,” CHD patients with diabetes have been clearly defined as very high-risk ASCVD patients, which is significantly different from CHD or diabetes alone. Therefore, CHD combined with diabetes can be studied as an independent population (9).

As previous evidence indicates the benefits of glycemic control for CVD, glycemic control has been assessed in current clinical management for CHD patients, using fasting plasma glucose (FPG) and glycated hemoglobin (HbA1c) levels (10). However, in some individuals, HbA1c does not match blood sugar levels and may be higher or lower than expected relative blood sugar levels (11). This inconsistent, between HbA1c levels and underlying glucose levels may limit the accuracy of HbA1c measurements in guiding treatment regimens. Non-glucose-related factors may affect the relationship between HbA1c levels and glucose levels. Although elevated glucose could be considered as a risk factor for CHD patients, it has limited predictive value for cardiovascular outcomes (12). HbA1c levels could reflect changes in blood glucose in patients over the past 8–12 weeks, and studies have shown that HbA1c levels are highly associated with the risk of CVD (13). However, with better understanding of HbA1c, researchers realized that HbA1c levels can be affected by multiple factors, such as blood sugar concentration, genetics and red blood cell life cycle, leading to inconsistency (10, 14). Therefore, HbA1c levels as a prognosis biomarker to assess is inaccurate for all populations. To improve the accuracy, the hemoglobin glycation index (HGI) was proposed by Hempe et al. (15) to quantify the change between measurements of HbA1c and mean plasma glucose level, a method that reflects the difference between actual HbA1c and predicted HbA1c based on FPG. Differences in the glycation of hemoglobin between individuals with the same FPG values can be assessed by the calculation of HGI. HGI is a measure of the difference between an observed HbA1c value and a predicted value based on blood glucose levels (16). Currently, some studies have shown that HGI has a predictive value for CVD in both type 2 diabetes mellitus (T2DM) and non-T2DM patients (17). However, no studies have evaluated the capacity of HGI to predict cardiovascular event risk in patients with CHD complicated with diabetes undergoing percutaneous coronary intervention (PCI) (18). Therefore, in this study, we aimed to evaluate the value of HGI in predicting major adverse cardiovascular events (MACE) in CHD complicated with T2DM patients undergoing PCI.

## Materials and methods

### Study design and population

This retrospective cohort study enrolled 1,050 patients with CHD complicated with T2DM who underwent PCI in the Department of Cardiology of the First Affiliated Hospital of Zhengzhou University, from December 2017 to December 2020. PCI operation and medication were performed in accordance with relevant guidelines (19). All patients were given aspirin and clopidogrel or ticagrelor preoperatively. The use of FFR, IVUS, OCT, and stent type is at the discretion of the clinician.

The inclusion criteria were as following: (1) patients were older than 18 years; (2) The patients met the diagnostic criteria of type 2 diabetes in the Chinese Guidelines for the Prevention and Treatment of Type 2 diabetes (2019 Edition) (20), in which T2DM was defined as FPG ≥ 7.0 mmol/L or 2 h plasma glucose oral glucose tolerance test ≥ 11.1 mmol/L or Typical symptoms of diabetes (eating, drinking, urinating, weight loss) and random plasma glucose ≥ 11.1 mmol/L; (3) At least one clinical phenotype of CHD, including non-ST segment elevation myocardial infarction (NSTEMI), ST segment elevation myocardial infarction (STEMI), unstable angina, and stable angina; (4)patients who were undergoing coronary angiography and according evidence of ischemia or hemodynamic related lesions received at least one stent implanted or balloon dilatation *via* PCI; (5) Complete clinical data with ongoing follow-up; and (6)Standardize medication according to the “Expert Consensus on the Application of Oral Hypoglycemic Drugs for Cardiovascular Disease Complicated with Diabetes.”

Exclusion criteria included: (1) Patients with comorbid malignancies or hematologic diseases; (2) Patients taking medications such as glucocorticoids and acetylsalicylic acid, which may affect HbA1c test results; (3) Congenital heart disease; and (4) Patients with incomplete clinical records.

**FIGURE 1:**
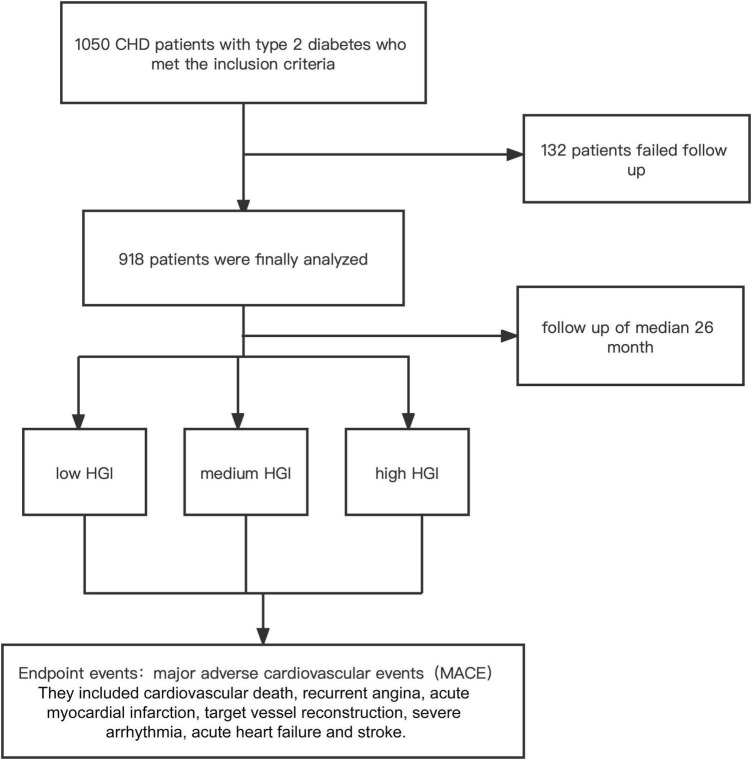
The follow chart of participants inclusion.

A total of 1,050 patients diagnosed with CHD combined with T2DM who underwent PCI were initially enrolled. However, 132 patients were lost contact due to the change in contact details during the follow-up period. As a result, data from 918 patients were analyzed. The study complied with the Declaration of Helsinki, and the protocol was approved by the ethics committee of the First Affiliated Hospital of Zhengzhou University. The detail of the design is registered on http://www.chictr.org.cn (identifier: ChiCTR-2200055450). Follow-up data were obtained by review of the medical records and/or telephone interview. The follow-up chart was illustrated in Figure 1.

### Clinical data collection

Data on clinical and demographic characteristics, including gender, age, drinking history, smoking history, Duration of diabets (years), medication history, and GRACE risk score, were collected from medical records. Fasting blood samples were drawn from each patient within 24 h after admission. Tosoh Automated Glycohemoglobin Analyzer (HLC-723G8, Tokyo, Japan) was used to measure the HbA1c levels. Stago autoanalyzer with the STA fibrinogen kit (Diagnostica Stago, Taverny, France) was used to measure the concentrations of plasma fibrinogen. The concentrations of fast blood glucose (FBG/FPG) were measured by the enzymatic hexokinase method. Other laboratory indices, including lipid profiles [total cholesterol (TC), triglyceride (TG), levelslow-density lipoprotein cholesterol (LDL-C), and high-density lipoprotein cholesterol (HDL-C)], estimated glomerular filtration rate (eGFR), creatinine, urea, D-Dimer were examined with standard biochemical techniques at the core laboratory in the First Affiliated Hospital of Zhengzhou University. According to modified Simpson’s rule, left ventricular ejection fraction (LVEF) was measured from two-dimensional echocardiography.

Laboratory indicators were included: FPG/FBG, HbA1c, Urea, D-Dimer, Fibrinogen, TC, LDL-C, HDL-C and TG levels, complete blood count, white blood cell (WBC) subset count, NT-pro BNP, and LVEF are also included.

### Follow up

All patients were followed up for a mean of 26 months after PCI *via* telephone, and outpatient clinic visits. The endpoint of this study was the occurrence of MACE. As a collective term, MACE included cardiovascular death, recurrent angina, acute myocardial infarction, target vessel reconstruction, severe arrhythmia, acute heart failure and stroke.

### Hemoglobin glycation index calculation

**FIGURE 2:**
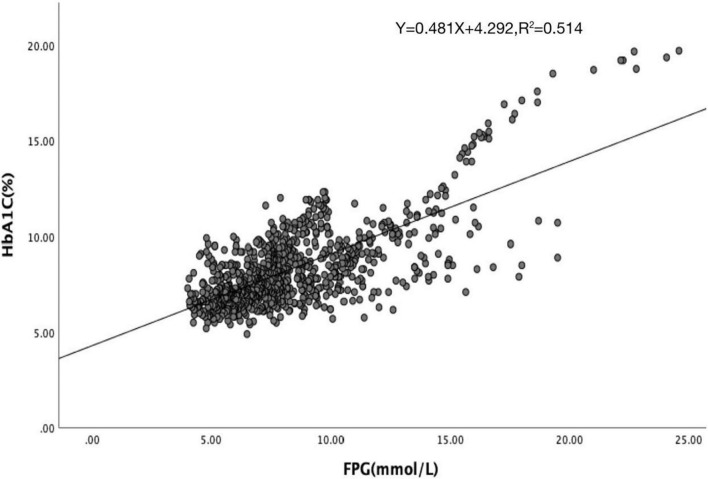
Correlation between HbA1c and FPG.

Hemoglobin glycation index was calculated by subtracting the predicted HbA1c from the observed HbA1c. The predicted HbA1c value was calculated by linear regression analysis with the FPG concentrations derived from study subjects. The linear relationship between HbA1c and FPG was estimated from the linear regression analysis of the subjects’ data. The scatter plot is obtained through the linear relationship between FPG and HbA1c (Figure 2). According to the results of the scatter plot, we removed 35 extreme outliers, and finally obtained the linear regression equation: predicted HbA1c = 0.481 × FPG + 4.292, *R*^2^ = 0.514, HGI = measured HbA1c–predicted HbA1c.

### Statistical analysis

All analyses were performed using SPSS 26.0 for windows statistical software (SPSS Inc., Chicago, IL, USA). Data were expressed as mean ± standard deviation (SD) or median ± interquartile range (IQR) for continuous variables, or percentages for categorical variables. Differences between normally distributed numerical variables between groups were analyzed by *T*-test, whereas non-normally distributed variables were analyzed by Mann–Whitney *U*-test. Comparison between the three groups was performed by one-way ANOVA. Categorical variables were summarized as percentages and compared using the chi-square (χ2) test. Correlation between HbA1c and FPG using Pearson’s linear equation. Multivariate Cox proportional hazards models were used to determine independent parameters of MACE. To construct the Cox model, univariate models for each of all predictor variables were conducted, and those variables that were significant (*P* < 0.05) in the univariate Cox model were then simultaneously entered into the multivariate Cox model. Hazard ratios (HRs) and 95% confidence intervals (CIs) were calculated. Cumulative survival curves for MACE were constructed using the Kaplan Meier method. *P* < 0.05 was considered as significant. Receiver operating characteristic (ROC) curve was performed to discuss the diagnostic value of risk factors for the prediction of poor prognosis. We use R language meter for model validation. The old model is GRACE risk score, and the new model is GRACE risk score combined with HGI. The cox regression model was constructed with the survival package and the net reclassification improvement/net reclassification index (NRI) values were calculated with the nricens package (21).

## Results

### Comparison of the clinical and laboratory characteristics according to the hemoglobin glycation index

**FIGURE 3:**
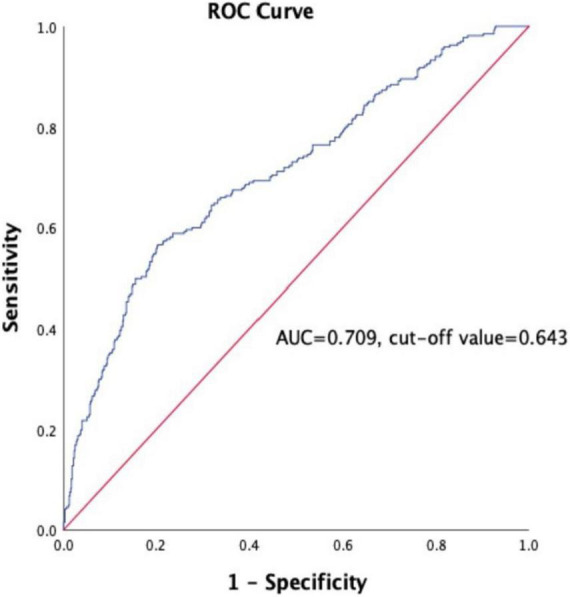
Receiver operating curve (ROC) for the analysis of HGI as the predictor of MACE in the study population. AUC indicates area under curve.

A total of 918 patients with CHD complicated with T2DM were included in this study. The median follow-up time was 26 months. The follow-up chart was illustrated in Figure 1. Pearson’s linear correlation analysis showed a linear regression correlation between HbA1c and FPG levels in the 918 patients included in this study (*R*^2^ = 0.514, *P* < 0.001). Through linear regression analysis, the regression equation for predicting HbA1c was calculated as predicting HbA1c = 0.481 × FPG + 4.292 (Figure 2). The study subjects were divided into 3 groups (27, 46, and 27%) based on HGI values: Low HGI (*n* = 219), with HGI ≤ −0.83; medium HGI (*n* = 422), −0.83 <HGI <0.91; and high HGI (*n* = 247), with HGI ≥ 0.91. According to receiver operating curve (ROC) analysis and Youden’s index, HGI level could predict MACE with a sensitivity of 56.6% and a specificity of 79.7% (cutoff value = 0.64, AUC = 0.709, *P* < 0.001) (Figure 3). In addition, high HGI patients had higher levels of Neut, Lymph, HbA1c, FPG, Diabetes Duration years, Fibrinogen, D-Dimer, and NT-pro BNP (*P* < 0.05), while LVEF (%) were decreased (*P* < 0.05), as shown in Table 1. A total of 267 cases accompanied with the occurrence of MACEs at an incidence rate of 29.08%. Therefore, patients were further divided into MACE (*n* = 267) and MACE-free (*n* = 651) groups. Clinical, echocardiographic, and laboratory data of the study population were shown in Table 2. Interestingly, factors such as the duration of diabetes, WBC, Nneut, TG, HbA1c, FPG, Fibrinogen, NT-Pro BNP, D-Dimer, Urea, GRACE risk score, and HGI ratios were higher in the MACE group than in the MACE-free group. On the contrary, the LVEF% ratio of the MACE group was lower than that of the MACE-free group (Table 2).

**TABLE 1:** Clinical and laboratory characteristics according to the HGI index.

Characteristics	Q1: Low HGI index (Group *n* = 249) HGI ≤ −0.83	Q2: Medium HGI index (Group *n* = 422) −0.83 < HGI < 0.91	Q3: High HGI index (Group *n* = 247) HGI ≥ 0.91	*P*
Age (years)	60.29 ± 10.47	59.91 ± 9.66	59.69 ± 11.11	0.809
**Gender**				
Male, *n* (%)	167 (67.1)	261 (61.8)	168(68.0)	0.193
Duration of diabetes (years)	6 (2–10)	6 (2–10)	7 (4–10)	0.019
**Laboratory parameters**				
WBC, 10^9^/L	7.67 ± 6.87	7.37 ± 3.89	7.94 ± 2.49	0.300
Neut, 10^9^/L	5.15 ± 2.59	4.82 ± 2.28	5.74 ± 2.66	<0.001
Lymph, 10^9^/L	1.81 ± 0.54	1.95 ± 0.62	1.87 ± 0.57	0.015
TC (mmol/L)	3.85 ± 1.37	3.65 ± 1.18	3.74 ± 1.04	0.133
TG (mmol/L)	1.67 ± 0.85	1.71 ± 1.07	1.78 ± 0.99	0.487
HDL-C (mmol/L)	1.02 ± 0.26	1.00 ± 0.24	0.98 ± 0.23	0.278
LDL-C (mmol/L)	2.17 ± 0.86	2.09 ± 0.90	2.20 ± 0.88	0.192
HbA1c (%)	7.12 ± 1.21	7.92 ± 1.35	10.98 ± 2.79	<0.001
FPG (mmol/L)	7.67 ± 2.68	9.42 ± 3.49	9.76 ± 4.46	<0.001
Urea (mmol/L)	5.60 (4.80–6.80)	5.72 (4.79–6.80)	5.74 (4.90–7.52)	0.363
Fibrinogen (g/L)	3.21 ± 0.79	3.09 ± 0.76	3.37 ± 0.95	<0.001
D-Dimer (mg/L)	0.09 (0.06–0.19)	0.09 (0.06–0.19)	0.13 (0.07–0.26)	0.002
LVEF (%)	60.33 ± 6.97	60.25 ± 6.02	58.37 ± 7.40	0.001
NT-pro BNP (pmol/L)	193.90 (78.32–689.00)	226.50 (83.82–728.95)	448.5 (114.67–1688.25)	<0.001
Daily smoke (%)	96 (38.5)	168 (75.7)	111 (44.9)	0.295
Daily drinken (%)	95 (38.2)	155 (36.7)	99 (40.1)	0.689
Aspirin (%)	238 (95.6)	401 (95.0)	234 (94.7)	0.905
Statins (%)	241 (96.8)	406 (96.2)	240 (97.2)	0.792
B eta blocker (%)	199 (79.9)	316 (74.9)	193 (78.1)	0.294
CCB (%)	85 (34.1)	126 (29.9)	77 (31.2)	0.512
Insulin (%)	64 (25.7)	86 (20.4)	64 (25.9)	0.153
Metformin (%)	128 (53.3)	211 (51.0)	122 (49.8)	0.727

WBC, white blood cell; Neut, neutrophils; Lymph, lymphocyte; TC, total cholesterol; TG, triglyceride; HDL-C, high density lipoprotein cholesterol; LDL-C, low-density lipoprotein cholesterol; HbA1c, glycosylated hemoglobin; FPG, fasting blood glucose; LVEF, left ventricular ejection fraction; CCB, calcium channel blocker.

**TABLE 2:** Clinical and laboratory characteristics according to the MACE.

Characteristics	MACE group (*n* = 267)	MACE-free group (*n* = 651)	*P*
Age (years)	60.29 ± 10.71	59.83 ± 10.10	0.53
**Gender**			
Male, *n* (%)	181 (67.8)	415 (63.7)	0.244
Duration of diabetes (years)	8 (3–10)	5 (2–10)	0.001
**Laboratory parameters**			
WBC, 10^9^/L	8.43 ± 4.76	7.27 ± 4.54	0.001
Neut, 10^9^/L	6.00 ± 2.87	4.81 ± 2.24	<0.001
Lymph, 10^9^/L	1.89 ± 0.59	1.89 ± 0.56	0.889
TC (mmol/L)	3.76 ± 1.18	3.70 ± 1.22	0.492
TG (mmol/L)	1.84 ± 1.04	1.67 ± 0.97	0.022
HDL-C (mmol/L)	1.01 ± 0.25	0.10 ± 0.24	0.454
HbA1c (%)	10.26 ± 3.16	7.81 ± 1.46	<0.001
FPG (mmol/L)	10.38 ± 4.32	8.01 ± 2.99	<0.001
Urea (mmol/L)	6.00 (4.90–7.26)	5.63 (4.79–6.83)	0.034
Fibrinogen (g/L)	3.38 ± 0.95	3.12 ± 0.77	<0.001
NT-pro BNP (pmol/L)	528.00 (163.00–1805.00)	190.30 (73.00–645.00)	<0.001
LVEF (%)	57.84 ± 7.74	60.56 ± 6.09	<0.001
D-Dimer (mg/L)	0.12 (0.07–0.27)	0.09 (0.05–0.19)	<0.001
Daily smoke (%)	108 (40.4)	267 (41.0)	0.874
Daily drinken (%)	98 (36.7)	251 (38.6)	0.600
HGI	0.94 (−0.42 to 2.16)	−0.28 (−1.06 to 0.44)	<0.001
GRACE risk score	126.03 ± 25.593	118.98 ± 23.961	<0.001

WBC, white blood cell; Neut, neutrophils; Lymph, lymphocyte; TC, total cholesterol; TG, triglyceride; HDL-C, high density lipoprotein cholesterol; HbA1c, glycosylated hemoglobin; FPG, fasting blood glucose; LVEF, left ventricular ejection fractions; HGI, hemoglobin glycation index.

### Univariate vs. multivariate cox regression analysis for the occurence of major adverse cardiovascular event

To identify independent predictors of MACE, we performed cox proportional hazards analysis to construct model 1 and model 2, which predict the risk factors of MACE for CHD patients with T2DM after PCI (Table 3).

**TABLE 3:** Univariate and multivariate cox regression analysis results for MACE.

Variables	Univariate analysis		Multivariate analysis			
	Crude HR	Crude	Model 1		Model 2	
	(95% CI)	*P*-value	Adjusted HR	Adjusted	Adjusted HR	Adjusted
			(95% CI)	*P*-value	(95% CI)	*P*-value
Duration of diabets	1.030 (1.012–1.049)	0.001	1.019 (1.000–1.039)	0.051	1.022 (1.003–1.042)	0.025
D-Dimer (mg/L)	1.037 (1.005–1.070)	0.022	0.981 (0.945–1.019)	0.321	1.018 (0.981–1.055)	0.349
Neut, 10^9^/L	1.105 (1.062–1.149)	<0.001	1.027 (0.983–1.078)	0.219	1.068 (1.021–1.117)	0.004
NT-pro BNP (pmol/L)	1.000 (1.000–1.000)	<0.001	1.000 (1.000–1.000)	0.015	1.000 (1.000–1.000)	0.002
Fibrinogen (g/L)	1.307 (1.150–1.486)	<0.001	1.125 (0.992–1.276)	0.066	1.129 (0.992–1.285)	0.067
LVEF (%)	1.006 (1.003–1.009)	<0.001	0.976 (0.959–0.993)	0.005	0.973 (0.956–0.989)	0.001
FPG (mmol/L)	1.121 (1.094–1.149)	<0.001	0.933 (0.888–0.981)	0.007		
HbA1c (%)	1.251 (1.206–1.298)	<0.001	1.406 (1.313–1.510)	<0.001		
HGI	1.531 (1.422–1.648)	<0.001			1.473 (1.365–1.589)	<0.001
GRACE risk score	1.010 (1.005–1.014)	<0.001	0.999 (0.994–1.004)	0.69	1.000 (0.996–1.005)	0.896

Neut, neutrophils; HbA1c, glycosylated hemoglobin; FPG, fasting blood glucose; LVEF, left ventricular ejection fractions; HGI, hemoglobin glycation index.

Univariate analysis showed that Duration of diabets (years) (HR: 1.030; 95% CI: 1.012–1.049, *P* = 0.001), D-Dimer (HR: 1.037; 95% CI: 1.005–1.070, *P* = 0.022), Neut (HR: 1.105; 95% CI: 1.062–1.149, *P* < 0.001), NT-pro BNP (HR: 1.000; 95% CI: 1.000–1.000, *P* < 0.001), Fibrinogen (HR: 1.307; 95% CI: 1.150–1.486, *P* < 0.001), LVEF (HR: 1.006; 95% CI: 1.003–1.009, *P* < 0.001), FPG (HR: 1.121; 95% CI: 1.094–1.149, *P* < 0.001), HbA1c (HR: 1.251; 95% CI: 1.206–1.298, *P* < 0.001), HGI (HR: 1.531; 95% CI: 1.422–1.648, *P* < 0.001), and GRACE risk score (HR: 1.010; 95% CI: 1.005–1.014, *P* < 0.001) were independent risk factors for MACE.

Collinearity analysis revealed that FPG and HbA1c had collinearity (VIF ≥ 5), and the rest collinearity analysis revealed no collinearity. After adjusting the covariates of Model 1, NT-pro BNP (HR: 1.000; 95% CI: 1.000–1.000, *P* = 0.015), LVEF (HR: 0.976; 95% CI: 0.959–0.993, *P* = 0.005), FPG (HR: 0.933; 95% CI: 0.888–0.981, *P* = 0.007), and HbA1c (HR: 1.406; 95% CI: 1.311–1.510, *P* < 0.001) were significant independent predictors of MACE.

Multivariate Cox analysis of Model 2 showed that Duration of diabets (years) (HR: 1.022; 95% CI: 1.003–1.042, *P* = 0.025), Neut (HR: 1.068; 95% CI: 1.021–1.117; *P* = 0.004), NT-pro BNP (HR: 1.000; 95% CI: 1.000–1.000, *P* = 0.002), LVEF (HR: 0.973; 95% CI: 0.956–0.989, *P* = 0.001), and HGI (HR: 1.473; 95% CI: 1.365–1.589, *P* < 0.001) were independent predictors of MACE.

### Receiver operating characteristic curve and risk of incident major adverse cardiovascular event according to hemoglobin glycation index

**FIGURE 4:**
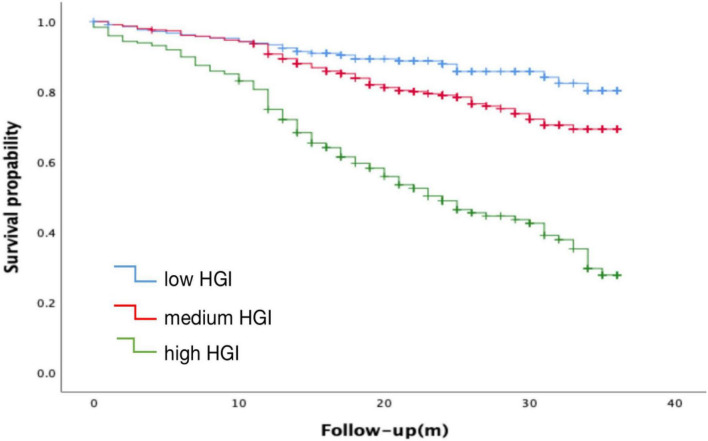
Kaplan-Meier estimates of the time to the first adjudicated occurrence of MACE.

According to receiver operating curve (ROC) analysis and Youden’s index, HGI level could predict MACE with a sensitivity of 56.6% and a specificity of 79.7% (AUC = 0.709, 95% CI: 0.616–0.747, *P* < 0.001), cut off value is 0.643, and Youden Index is 0.363 (Figure 3). The long-term survival of patients was analyzed by Kaplan–Meier survival analysis. There were significant differences in 3-year MACE among the three groups, as shown in Figure 4. Survival of patients declines as HGI levels rise. The incidence of MACE was higher in the high HGI group (log rank, *P* < 0.001). The GRACE score established by the Global Registry of Acute Coronary Events (GRACE) study is an important tool commonly used in clinical practice to assess the risk of hospitalized events in patients with ACS. We use R language meter for model validation. Comparing the old and new models according to the formula results in an NRI value of 26.3%. All values >0, indicating that the diagnostic accuracy of the joint model is improved.

## Discussion

This study investigated the correlation between HGI and MACEs by analyzing 918 patients with CHD combined with T2DM undergoing PCI. Our study found that HGI was an independent predictor of MACE. More importantly, high HGI group had a higher risk to have MACE events with a significantly reduced survival rate.

Blood glucose levels in patients with type 2 diabetes can significantly affect the occurrence and prognosis of cardiovascular complications (12, 22). At present, the clinical indicator used to monitor the blood sugar level of patients is HbA1c, which can reflect the blood sugar level of patients in the past 8–12 weeks (13). However, recent studies have shown that HbA1c is not only related to blood sugar levels but also to diseases such as hemoglobinopathies, uremia, and hemochromatosis. Due to biological differences between individuals, fasting blood glucose is not an independent factor affecting HbA1c (10, 23, 24). Studies have found that the level of HbA1c in some patients is inconsistent with the level of blood sugar control, impacting the clinical prognosis evaluation based on this indicator (14, 25).

To address the issue, Hempe (15) and his group proposed the concept of HGI in 2002 to quantify differences in HbA1c among individuals. HGI was proposed to measure the deviation of glycated HbA1c from its expected value. A high HGI indicate an elevated level of hemoglobin glycation. As a newly proposed indicator to measure the degree of HbA1c control in recent years, HGI has been carried out in many studies and confirmed that HGI is independently related to the microvascular and macrovascular complications of diabetes (26). In addition, based on data from the prestigious American Cardiovascular Risk in Diabetes Study (ACCORD), Hempe et al. (27) found that the high HGI group had a higher rate of cardiovascular complications. Similarly, Van Steen et al. (28) found that adverse cardiovascular and cerebrovascular events and all-cause mortality were significantly lower in the low HGI group, and increasing HGI levels indicated high risk of cardiovascular and cerebrovascular mortality, according to RRAR agonist outcome data from a large study (Alecardio). In addition, Kim et al. (29) found that high HGI levels increase the risk of cardiovascular events by following a 10-year follow-up of patients with T2DM. Cheng et al. (12) found that compared with the low HGI group, T2DM patients in the high HGI group had a 2.9-fold increased risk of CHD. Other studies have shown that the higher the proportion of insulin use in diabetic patients, the longer the course of the disease, the longer the blood vessels are affected by hyperglycemia, the more serious the damage, thereby increasing the risk of CVD (30). These results all demonstrate the ability of HGI to predict cardiovascular complications in T2DM patients.

At present, the specific mechanism of correlation between HGI, diabetic, and CVD remains elusive. Some studies indicated advanced glycation end products (AGEs) would be one of the main contributor (24, 31). AGEs are intermediates in response to chronic hyperglycemic states. In addition to AGEs, several studies have shown that HGI levels are significantly correlated with C-reactive protein and inflammatory markers produced by polymorphonuclear leukocytes in the body (32). These results suggest that diabetic patients with high HGI levels have a high inflammatory state in the body, leading to greater damage to vascular endothelial cells, which in turn increases the risk of CHD or ischemic stroke (33). Interestingly, some studies also indicated the interaction between HGI and insulin plays an important role (34). Patients with high HGI use insulin to control blood sugar at a higher rate, indicating lack of capacity to control blood sugar well and the development of hyperglycemia, which negatively affected the function of various systems in the body (35, 36). Often high level associated oxidative stress associated contributes to the glycemic variability and poor prognosis. However, whether hypoglycemia induced high HGI levels increase cardiovascular risk is still debatable (37).

Our study confirms the prognostic value of HGI in CHD complicated with diabetes undergoing PCI. In our study, we identified the differences in related indicators at different HGI levels in CHD patients with diabetes mellitus. Our results showed that the elevated level of HGI was associated with increasing Neut, Lymph, HbA1c, FPG, Diabetes Duration years, Fibrinogen, D-Dimer, and NT-pro BNP (*P* < 0.05), while LVEF (%), were decreased (*P* < 0.05). Further multivariate regression analysis showed that high HGI level, high WBC count, high NT- proBNP, and low LVEF% were independent risk factors for MACE. Discordance between HbA1c and blood glucose has been reported, with many people consistently having HbA1c levels above or blow fasting blood glucose (11, 38), average blood glucose (self-monitoring) (38), or continuous glucose monitoring. We further calculated HGI through the linear equation relationship between FPG and HbA1c. Differences in the glycation of hemoglobin between individuals with the same FPG values can be assessed by the calculation of HGI. HGI is a measure of the difference between an observed HbA1c value and a predicted value based on blood glucose levels (26). The ROC curve was used to analyze the predictive value of HGI for CHD with MACE, the area under the curve was 0.709, the predictive sensitivity was 56.6%, and the specificity was 79.7%. At the same time, Kaplan-Meier survival analysis showed that the survival of patients in the high HGI group was significantly declined. The GRACE risk score is an important scoring tool recommended by the current domestic and foreign guidelines for ACS risk stratification and assessment of the risk of inpatient events (39, 40). However, when the GRACE risk score is used alone, its predictive value is relatively limited (41). In recent years, some studies have reported that the combination of some biochemical indicators with the GRACE risk score has a certain application value in evaluating the prognosis of patients with CHD (42). In this study, the model validation of GRACE risk score combined with HGI was established, and the NRI was 26.3%, indicating that when HGI combined with GRACE risk score, the predictive value of MACE events in patients with CHD complicated with T2DM can be further improved compared with the use of GRACE score alone. Taken together, HGI has a good clinical diagnostic value in assessing the occurrence of MACE in CHD patients with T2DM.

Despite demonstrating diagnostic potential of HGI, this study still has certain limitations. Ethnicity was limited to the Asian population due to the specificity of enrolled patients. Ethnicity is an important factor in HGI studies as the ACCORD study indicates that non-Caucasians have a higher HGI level as compared to other ethnicity (27). Also similar studies demonstrated that Hispanics, Asians and Africans have higher HbA1c levels compared to Caucasians (43). Due to the limited number of included studies, meta-regression and dose-response meta-analyses were not performed in this study. That being said, our future work will include multicenter and meta-analyses, including ethnicity, and the ability to guide clinical treatment based on HGI levels.

## Conclusion

In conclusion, our findings demonstrated the potential of HGI as an independent predictor of MACE events in patients with CHD complicated with T2DM undergoing PCI. HGI can be used for personalized assessment and prediction of cardiovascular adverse events.

## Data availability statement

The original contributions presented in this study are included in the article/supplementary material, further inquiries can be directed to the corresponding authors.

## Author contributions

JT and JZ conceived and participated in the study design and drafting of the initial manuscript. SX and ZQ contributed to the data acquisition, analysis, and drafted the manuscript. All authors contributed to the interpretation of the data, critically revised the manuscript, and approved the final version before submission.
